# How Age, Cognitive Function and Gender Affect Bimanual Force Control

**DOI:** 10.3389/fphys.2020.00245

**Published:** 2020-03-27

**Authors:** Julian Rudisch, Katrin Müller, Dieter F. Kutz, Louisa Brich, Rita Sleimen-Malkoun, Claudia Voelcker-Rehage

**Affiliations:** ^1^Institute of Human Movement Science and Health, Faculty of Behavioral and Social Sciences, Chemnitz University of Technology, Chemnitz, Germany; ^2^Department of Neuromotor Behavior and Exercise, Institute of Sport and Exercise Sciences, University of Muenster, Muenster, Germany; ^3^Department of Pediatrics, Technical University Munich, Munich, Germany; ^4^CNRS, Institute of Movement Sciences, Aix-Marseille Université, Marseille, France

**Keywords:** interlimb coupling, interhemispheric interference, bimanual force control, mild cognitive impairment, aging, corpus callosum, detrended fluctuation analysis

## Abstract

Coordinated bimanual control depends on information processing in different intra- and interhemispheric networks that differ with respect to task symmetry and laterality of execution. Aging and age-related cognitive impairments, but also sex can have detrimental effects on connectivity of these networks. We therefore expected effects of age, cognitive function and sex on bimanual force coordination. We furthermore expected these effects to depend on the characteristics of the task (i.e., difficulty and symmetry). 162 right handed participants (19 younger adults [YA], 21–30 years, 9 females; 52 cognitively healthy older adults [HOA], 80–91 years, 32 females; and 91 older adults with mild cognitive impairments [MCI] 80–91 years, 37 females) performed isometric bimanual force control tasks that required following constant or alternating (cyclic sine-wave) targets and varied in symmetry, i.e., (i) constant symmetric, asymmetric [with constant left and alternating right (ii) or vice versa (iii)], (iv) alternating in- and (v) alternating antiphase (both hands alternating with 0° or 180° relative phase, respectively). We analyzed general performance (time on target), bimanual coordination as coupling between hands (linear correlation coefficient) and structure of variability (i.e., complexity measured through detrended fluctuation analysis). Performance and coupling strongly depended on task symmetry and executing hand, with better performance in symmetric tasks and in asymmetric tasks when the left hand produced a constant and the right hand an alternating force. HOA and MCI, compared to YA, showed poorer performance (time on target) and reduced coupling in in- and antiphase tasks. Furthermore, both groups of OA displayed less complex structure in alternating force production tasks, a marker of reduced control. In addition, we found strong sex effects with females displaying reduced coupling during in- and antiphase coordination and less complex variably structure in constant force production. Results of this study revealed strong effects of age, but also sex on bimanual force control. Effects depended strongly on task symmetry and executing hand, possibly due to different requirements in interhemispheric information processing. So far, we found no clear relationship between behavioral markers of bimanual force control and age-related cognitive decline (compared to healthy aging), making further investigation necessary.

## Introduction

Many daily bimanual tasks require precise force adjustments for object stabilization and manipulation, e.g., when buttoning shirts or tying laces. Fine motor skills are thus an important factor for independent living. They are, however, often compromised in late adulthood (Vieluf et al., 2013). Bimanual task demands can add a further challenge to fine motor control, as they require precise spatiotemporal coordination of both hands. Different task-dependent intra- and interhemispheric neural networks [e.g., involving the primary motor cortex (M1), supplementary motor area (SMA), and premotor cortex (PMC), the cingulate and posterior parietal cortex; Swinnen, 2002] are necessary for controlling bimanual movement tasks. Connection between the two hemispheres is primarily established via the corpus callosum (CC) (Gooijers and Swinnen, 2014).

The pathways of information processing for bimanual control are highly dependent on the task symmetry, i.e., whether the task requires activation of homologous muscles in both limbs at the same time. In such symmetric tasks, a tight spatiotemporal coupling of hands (e.g., in- or antiphase) is required (Liuzzi et al., 2011), so that both hands are controlled in stable bimanual synergy (Kelso and Schöner, 1988). However, bimanual movement tasks in daily life are often asymmetric; the non-dominant hand usually takes a more static role (i.e., stabilizing) and the dominant hand a more dynamic role (i.e., manipulating) (Guiard, 1987). In this case, when asymmetric (or disparate) movements are performed with both hands, active inhibition of the spontaneous coupling (due to cross-talk) of the individual limbs commands is necessary (Daffertshofer et al., 2005). For example, Grefkes et al. (2008) have observed a positive interhemispheric modulation of exchanged information (i.e., active facilitation) during symmetric bimanual movements and a negative modulation (i.e., inhibition of cross-talk) during unimanual movements. In addition to requiring interhemispheric inhibition, task asymmetries are expected to result in the development of specialized lateralization of the hands (Guiard, 1987; Wang and Sainburg, 2007) and hemispheric asymmetries (van den Berg et al., 2010). In this vein, higher inhibition of non-isodirectional movements have been shown in the non-dominant hemisphere (i.e., the right hemisphere in right-handers) as compared to the dominant (Wenderoth et al., 2004; van den Berg et al., 2010).

Previous behavioral studies have shown bimanual coordination difficulties in older adults (OA). For example, Temprado et al. (2010) revealed that antiphase bimanual coordination modes are less stable in OA as compared to younger adults (YA). Furthermore, Bangert et al. (2010) found less accurate timing in asynchronous bimanual tasks in OA than YA. These alterations have been attributed to structural and functional changes in the aging brain including areas that are of relevance in bimanual coordination (for review, see Maes et al., 2017). Using structural magnetic resonance imaging, widespread gray (Giorgio et al., 2010) and white (Pagani et al., 2008) matter deteriorations have been shown with aging, with earlier onset and pronounced progression in frontal areas of the brain. Functionally, OA have been shown to display bilateral hyper-activation, for example, in the SMA or the secondary somatosensory cortex (Goble et al., 2010) as compared to YA when performing in- and antiphase wrist flexion tasks. Positive correlations between brain activation and performance are interpreted as hyper-activations, i.e., a compensatory mechanism as a result of reduced interhemispheric inhibition (Goble et al., 2010; Maes et al., 2017).

In addition to the effects of reduced interhemispheric inhibition, bimanual coordination can be severely affected when interhemispheric connectivity is disrupted. As opposed to reduced inhibition, however, changes to interhemispheric connectivity also affect symmetric movement tasks and asymmetric task performance can potentially be improved due to a lack of cross-talk (Kennerley et al., 2002). Individuals that present age-related cognitive declines [e.g., mild cognitive impairments (MCI) or Alzheimer’s dementia] show an even larger reduction of the connectivity between intra- and interhemispheric neural networks (Frederiksen, 2013; Stricker et al., 2016). Such a “functional disconnection” (O’Sullivan et al., 2001) can add further challenges to the control of bimanual movement tasks for older adults within even impaired interhemispheric inhibition. Other possible consequence of this disconnection may be that information processing is less distinct, and/or that additional networks in the brain take over the function of highly specialized areas (Voelcker-Rehage and Niemann, 2013). Even though the importance of cognitive functioning for bimanual control has been emphasized (Swinnen and Wenderoth, 2004), the impact of cognitive impairment on bimanual coordination has not been yet investigated.

Apart from an impact of age and cognitive functioning on interhemispheric neural networks, previous studies have also reported sex differences with a greater relative callosal area of women than men in the splenium of the CC (Davatzikos and Resnick, 1998), but with age having a stronger impact on CC diffusivity in women than men (Sullivan et al., 2010). Only few studies have investigated sex effects on bimanual coordination, those who did reported slight female disadvantages (Mickeviciene et al., 2011; Shetty et al., 2014). To the best of our knowledge, no study has investigated the interaction between age and sex on bimanual coordination including bimanual force control.

Human motor performance is based upon multilevel control processes including sensorimotor control and cognition as well as cardiovascular capacities and biomechanical properties. Better understanding the physiological and pathological moderating factors of bimanual force control goes through recognizing the involvement of the interaction of such multilevel control processes. This fact, generalizable also to other physiological processes, has led to a growing interest in adopting a complex systems approach to study human performance and aging (Goldberger, 1996; Goldberger et al., 2002a; Lipsitz, 2002; Sleimen-Malkoun et al., 2014). Accordingly, scientists have started to focus on macroscopic markers extracted from physiological and behavioral signals fluctuations. These markers are considered to reflect the intricate interactions between the different functional processes and to relate to the overall system’s adaptability (Lipsitz and Goldberger, 1992; Vaillancourt and Newell, 2003; Vieluf et al., 2015; Knol et al., 2019; Torre et al., 2019), with more irregular signals over time, termed as complex, reflecting richer functional interactions and better capacities to adapt. To do so one needs to go beyond the standard statistical measures of variability (e.g., the standard deviation of a time series) that average out the time-structure of the expressed fluctuations. In this regard, one of the widely used measures is detrended fluctuation analysis (DFA; Peng et al., 1995) that computes self-similarity of biological, not necessarily stationary, signals on different time scales. Assuming the presence of long-range time auto-correlations, DFA analyses the relationship between the amplitude of the fluctuations and the time window width (defining a given time-scale) at which they were measured. This relationship yields a scaling exponent (α) that needs to be compared to that of known, more or less random, complex, or strongly deterministic processes, in order to characterize the process underlying the studied empirical signal. Applying this method, and other non-linear metrics, in the context of force control and aging, Vaillancourt and Newell (2003) have shown that OA (despite higher overall variability) display a more regular variability time-structure during a unimanual constant force production task compared to YA; thus their behavior was considered as less complex. This age-related loss of complexity was not observed, however, when the task consisted in producing a regular force pattern (tracking a sine-wave). Here, OA showed more complex patterns than YA. A similar result was also reported by Knol et al. (2019), reinforcing the suggestion that OA are disadvantaged in terms of adequately modulating their neuromuscular degrees of freedom (DOF) in response to task constraints (Vaillancourt and Newell, 2003). Less is known, however, about behavioral complexity in bimanual tasks of varying asymmetry and whether similar age-related changes in complexity (i.e., the structure of variability) can be observed irrespective of the motor task (symmetric or asymmetric task) and the cognitive status of participants.

The aim of this study was to investigate behavioral signatures of bimanual force control in tasks that vary in difficulty and symmetry (cf. Table 1). Further, we wanted to investigate whether executing hand, age, age-related cognitive impairments and sex had differential impact on these measures as expected due to differential effects of these factors to intra- and interhemispheric information processing. To date, the effect of age and cognitive status on bimanual coordination has not been systematically tested when tasks require different contributions of interhemispheric inhibition or facilitation (i.e., reflecting the different bimanual task demands in daily life). Investigating differential effects in task performance with respect to age might help to early diagnose age-related cognitive pathological decline and to differentiate aging and disease. We hypothesized that, in addition to the effects of healthy aging, pathological age-related cognitive decline would have a detrimental effect on bimanual performance, particularly when strong interhemispheric coupling is required. That is, healthy OA (HOA) may display performance deteriorations in asymmetric bimanual tasks, as has been shown in previous studies (Bangert et al., 2010; Temprado et al., 2010). As opposed to that, in OA with mild cognitive impairments, interhemispheric connectivity might be reduced as a consequence of structural deteriorations in the area of the CC. This may, on the one hand lead to poorer performance in tasks where close coupling of the two hands is required; but, on the other hand, might have a preservative function for performance during asymmetric tasks due to a reduction of interhemispheric cross-talk. We furthermore explored how performance interacted with age and sex due to previously reported differences in CC development between men and women (Davatzikos and Resnick, 1998; Sullivan et al., 2010). We therefore analyzed different measures reflecting performance with respect to the general task performance (i.e., TOT), behavioral complexity as it can be inferred from the time-structure of force control signals (i.e., DFA) as well as interlimb coupling (i.e., BCC).

**TABLE 1:** Classification scheme for the tasks by their difficulty (rows) and whether interhemispheric inhibition is required (asymmetric tasks) or not (symmetric tasks).

Difficulty Inhibition Required	Low	Medium	High
No	Constant symmetric (i)	Alternating inphase (iv)	
Yes	Constant asymmetric (ii, iii)	Alternating asymmetric (ii, iii)	Alternating antiphase (v)

## Materials and Methods

### Participants

**FIGURE 1:**
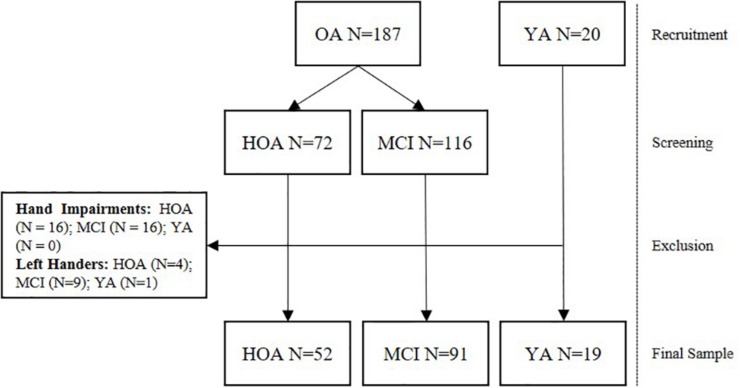
Flow chart displaying the number of participants recruited, screened and excluded from the study.

Data from a total of 162 participants from three different groups consisting of 19 YA (21–30 years, 9 female), 52 HOA (80–91 years, 32 female), and 91 MCI (80–91 years, 37 female) were analyzed in this study (see Table 2 for detailed group characteristics and Figure 1 for a flow chart of the recruitment and screening procedures). Older adults were recruited as part of the Sensor-based Systems for Early Detection of Dementia (SENDA) project (Müller et al., 2020) and allocated to the HOA or MCI group after screening (see section “Cognitive Screening”). The study was advertised with local general practitioners and in newspapers. Older adults from 80 years of age that did not show any severe motor impairments, any presence of psychiatric, neurocognitive or neurological disorders; any presence of severe cardiovascular, pulmonary or musculoskeletal disorders; any presence of diabetic neuropathy; any substance abuse were included in this study. Participants needed to be able to understand and follow the instructions. Participation in clinical trials including anti-dementia drugs was not permitted during the time of the study. Finally, capability of traveling independently to the laboratory premises was required for this study. OA received monetary compensation of 20 € for their participation Participants in the group of YA were recruited from the body of students at the Institute of Human Movement Science and Health at Chemnitz University of Technology, Germany and received credits for their study program instead of monetary compensation. For this study, all left-handers (*n* = 15) and participants with existing conditions affecting fine motor skills of their hands (e.g., arthritis, *n* = 32) were excluded.

**TABLE 2:** Participant group characteristics.

	YA	HOA	MCI	Group Differences			
				
				ANOVA	YA vs. HOA	YA vs. MCI	HOA vs. MCI
N	19	52	91		−	−	−
Age (years)	21.0 (2.6)	82.3 (2.4)	82,7(2.3)	*F*(2,158) = 5182, ***p* < 0.001**	***p* < 0.001**	***p* < 0.001**	*p* = 0.400
Years of Education	16.2 (2.7)	14.4 (3.0)	13.7 (3.3)	*F*(2,158) = 5.22, ***p* = 0.006**	***p* = 0.073**	***p* = 0.005**	*p* = 0.175
Height (cm)	176.4 (2.0)	167.1 (2.3)	167.8 (1.7)	*F*(2,159) = 11.77, ***p* < 0.001**	***p* < 0.001**	***p* = < 0.001**	*p* = 0.340
Weight (Kg)	72.5 (11.1)	73.9 (9.1)	74.9 (13.1)	*F*(2,159) = 0.37, *p* = *0.695*	−	−	−
BMI (m/kg^2^)	23.1 (2.7)	27.3 (3.5)	27.0 (3.7)	*F*(2,159) = 11.00, ***p* < 0.001**	***p* < 0.001**	***p* < 0.001**	*p* = 0.570
MVC (N)	66.7 (29.7)	56.0 (17.0)	57.2 (19.3)	*F*(2,159) = 2.11, *p* = 0.124	−	−	−
FM use	15.2 (2.2)	13.2 (3.2)	11.9 (3.6)	*F*(2,153) = 8.11, *p* < 0.001	*p* = 0.05	*p* < 0.001	*p* = 0.05
Sex (m/f)	10/9	20/32	54/37	*X ^2^*(2) = 5.78, *p* = 0.060	−	−	−

*Count data are presented for N, Sex, and Handedness; Mean (Standard Error) for age, height, weight, BMI, years of education, and MVC of the participants. *P*-values for between group differences are derived from *t*-tests. YA, younger adults; OA, older adults; MCI, older adults with mild cognitive impairments; MVC, maximum voluntary contraction; BMI, body mass index; m, male, f, female, FM use, frequency of performing different tasks (answered on a 5-point Likert scale for each task) that require fine motor skills (i.e., typing on a keyboard, writing, needlework or model building). Bold values indicate significant effects at *p* < 0.05.*

The study was approved by the Research Ethics Committee at the faculty of Behavioral and Social Sciences at Chemnitz University of Technology (V-232-17-KM-SENDA-07112017). Participation in the study was voluntary and individuals that wished to participate signed their consent after reading a detailed participant information sheet and were the opportunity to ask questions.

### Cognitive Screening

**FIGURE 2:**
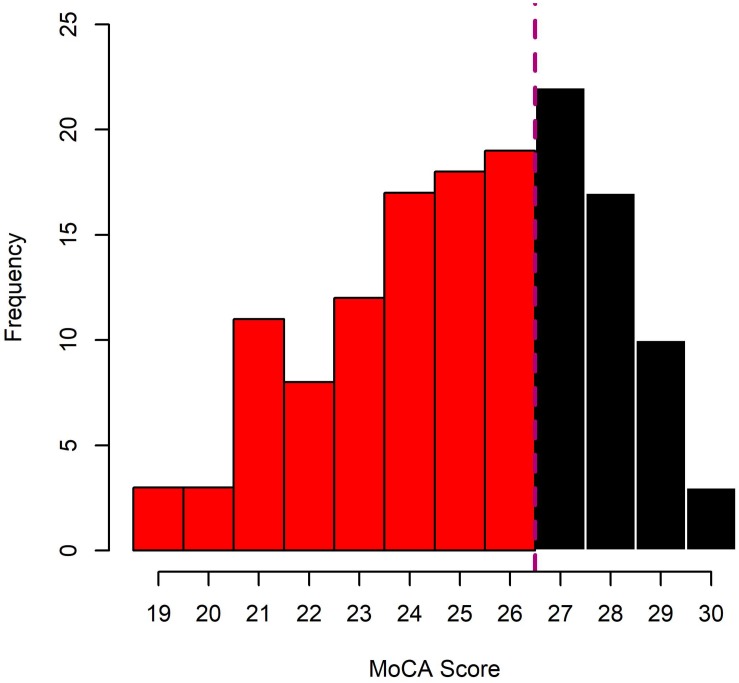
Distribution of MoCA scores in the sample of cognitively healthy older adults (HOA; black bars) and older adults with mild cognitive impairments (MCI; red bars).

Prior to their group allocation, HOA and MCI underwent a cognitive screening procedure, using the Montreal Cognitive Assessment (MoCA). As originally proposed (Nasreddine et al., 2005) individuals with MoCA scores of 26 or lower were allocated to the MCI group, individuals above 26 to HOA (see Figure 2 for distribution of MoCA Scores across HOA and MCI). Individuals with scores of 18 or lower were excluded since more severe cognitive impairments were likely to be present.

### Further Screening and Handedness

Additional questionnaires were included in the screening process to assess demographic information, education level (years of education), subjective health status and frequency of hand use during manual activities of daily life (cf. Table 1). Handedness of the participants was assessed prior to the experimental tasks using the Edinburgh handedness inventory (Oldfield, 1971). Only right-handed participants were included in the analysis.

### Apparatus

**FIGURE 3:**
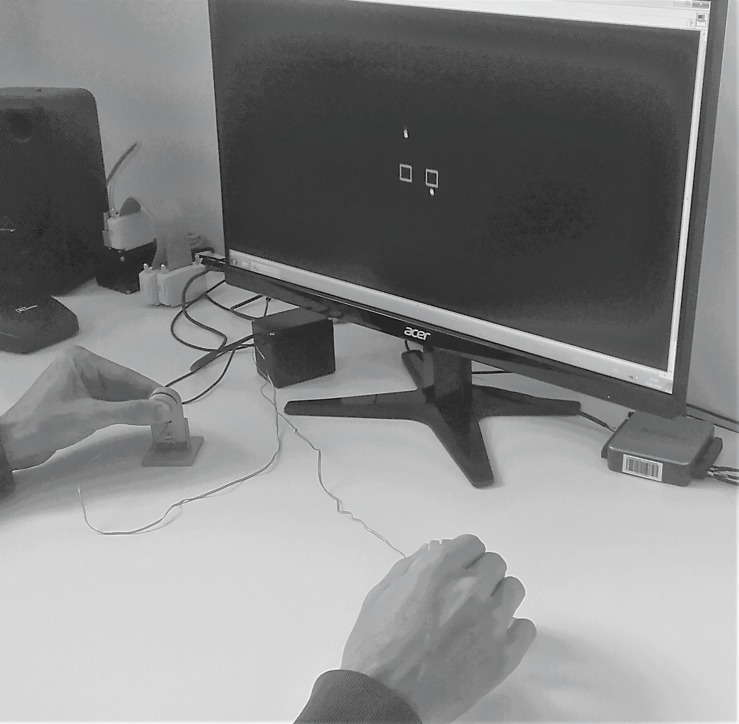
Participant’s point of view during execution of the experimental procedure. The participant applies force to the two force transducers using a pinch grip with thumbs and index fingers of both hands. Feedback of the magnitude of his/her applied force is given by the yellow dots on the screen. The square boxes indicate the reference force levels.

Force was measured using two compression load cells with a diameter of 29.5 mm, a depth of 8 mm and a measurement range of 0 – 22.5 N (Manufacturer: Measurement Specialties Inc., Hampton, VA, United States; Model: FX-1901-0001-50L) (cf. Hübner et al., 2018 for comparable unimanual setup). Signals were pre-amplified (using a customized voltage amplifier), digitally converted and sampled at a frequency of 120 Hz, using a NI-DAQ USB-6002 (National Instruments, Austin, TA, United States). Experimental procedures, i.e., data acquisition and real time visual feedback, were programed using a customized LabView 2015 (National Instruments, Austin, TA, United States) script. Force transducers were placed in front of the participants at a comfortable position. Participants were seated at a distance of 60 cm in front of a 23.8 inch monitor (hardware resolution 1920 × 1080 pixels) which provided real-time feedback (updating frequency of the screen was 60 Hz) about actual force levels of the participants and target forces that need to be met (see Figure 3). Feedback about the magnitude of the applied force to both sensors was indicated by two small dots that moved up (i.e., larger forces) and down (i.e., smaller forces). Small square-shaped rectangles (width and height of 12.5 mm) moving up and down were shown on each side as well, indicating reference values (see Figure 2). The scale of the display was adjusted with respect to individual force ranges and the size of the target box corresponded to 3% of MVC.

### Procedures

Prior to the experiment, participants were instructed to hold the two sensors using a pinch grip between their thumb and index fingers and without lifting the sensors off the table. Further, they were asked to rest their forearms comfortably on the table. Initially, maximum voluntary contraction (MVC) of the dominant hand was determined to adjust force ranges individually during subsequent tasks. To determine MVC, participants were asked to apply as much force as possible to the transducer without changing the instructed pinch grip for a duration of 5 s. The procedure was repeated three times in a row (with a rest period of about 10 s in between) and the largest peak value measured during all three trials was used as individual MVC.

Participants then performed different bimanual experimental tasks using two force transducers. Each hand was either required to apply a constant force or an alternating force pattern that followed a sine-wave form. Five different bimanual conditions were tested based on the assumption that they differ in difficult level and require different amounts of interhemispheric inhibition (see Table 1 for classification regarding general difficulty and inhibition required). Tasks included producing (i) a constant force applied by both hands at 12% of MVC (*constant symmetric*); an asymmetric pattern with either the non-dominant (ii) or the dominant hand (iii) applying a constant force (*constant asymmetric*) at 12% of MVC and the opposite hand producing an alternating sine-wave force pattern (*alternating asymmetric*) between 5 and 12% of MVC (*asymmetric with non-dominant [dominant respectively] constant*); (iv) an alternating symmetric sine-wave force pattern produced by both hands with a relative phase of 0° between hands and a force range between 5 and 12% of MVC (*alternating inphase*); and (v) an alternating asymmetric sine-wave pattern produced by both hands with a relative phase of 180° and a force range between 5 and 12% of MVC (*alternating antiphase*).

Frequency of the sine wave in all alternating conditions was 0.2 Hz and each task had a duration of 20 s (resulting in a total of four sine waves per trial in the alternating force conditions). Participants completed eight trials per condition. Only two trials were performed in the constant symmetric condition due to ease of the task and to avoid tiring of the participants (in view of the additional procedures the participants underwent on the same day). Participants practiced three inphase and four antiphase trials initially to familiarize with the task. All participants performed the task in the same order (1) *Alternating inphase*, (2) *Alternating antiphase*, (3) *Constant symmetric*, (4) *Asymmetric with left hand*, and (5) *Asymmetric with right hand constant*. Overall, participants required roughly 30 minutes to complete the task.

### Data Analysis

Pre-processing of the data as well as statistical analysis was performed using the R 3.4.4 base package (R Core Team, 2017). The additional modules “non-linearTseries” (Garcia, 2018) and “nlme” (Pinheiro et al., 2018) were used for Detrended Fluctuation Analysis and statistical modeling, respectively.

#### Pre-processing and Outcome Variables

The initial 5 s of each trial (relating to one full cycle in the sine-wave patterns) were excluded from the analysis to ensure the participants had enough time to align their force with the reference. Three outcome variables were calculated that are indicative of (i) task performance; (ii) structure of variability; and (iii) bimanual coupling.

Task performance was measured as time on target (TOT), i.e., the amount of time (in %) participants kept their force within the target range of 0.6 % of MVC (upper and lower boundaries of the box).

Structure of force signal variability underlying force control was estimated using DFA (Peng et al., 1995). A scaling exponent (DFA-α) was calculated by (i) integrating the signal; (ii) dividing the integrated signal in non-overlapping segments; (iii) calculating the error of the least squares linear regression model for every segment (i.e., local trend); and (iv) computing square-root of the average error for all segments. These steps were repeated for different segment lengths. Segment lengths were a set of exponentially increasing numbers between a range of 10 and 200. Range of segment lengths was based upon previous work by Vaillancourt and Newell (2003) who have likewise calculated DFA for a constant and sine-wave force production task. We have increased our upper limit, however, as our sine-wave task required slower oscillations. Finally, a regression model was computed for the log-log relationship between the scaling factor and the fluctuation magnitude. The scaling exponent DFA-α (i.e., the regression parameter from this model) is indicative of the self-similarity properties of the signal, namely, how slowly autocorrelations decay across timescales. Accordingly, uncorrelated random signals yield lower DFA-α (about 0.5 for white noise), whereas highly structured and positively self-correlated signals yield higher DFA-α (about 1.5 in case of a Wiener process/Brownian noise). Complex signals, representing a compromise between randomness and predictably, like pink noise, have a power law decaying auto-correlations with DFA-α≈ 1. DFA-α values between 0 and 0.5 indicate negatively self-correlated signals (e.g., large fluctuations are followed by smaller fluctuations in time).

Bimanual coupling was assessed by a bimanual coupling coefficient (BCC) calculated as the linear correlation coefficient between dominant and non-dominant force signals. BCC > 0 (i.e., closer to 1) are indicative of inphase coupling of the two hands, coefficients < 0 (i.e., closer to −1) are indicative of antiphase coupling. Decoupling of the hands would result in coefficients close to 0. Presence of interhemispheric cross-talk (less interhemispheric inhibition) would result in higher (positive) correlation coefficients in the *asymmetric* and *antiphase* conditions (Zielinski et al., 2017).

#### Statistical Analysis

Linear mixed effects modeling was performed to investigate the effect of the main factors group, sex, executing hand and condition as well as the interaction between these factors on the different outcomes TOT, DFA-α, and BCC. F-statistics are reported for main effects. Partial eta-squared (η_*P*_^2^) is reported for effect size. Upon reaching significance (*p* < 0.05), planned model contrasts were further inspected. In the case of group comparisons, both YA and MCI were compared to the HOA group. In case of significant condition effects, successive contrast coding was used such that each condition was compared to the subsequent condition in order: *constant symmetric, constant asymmetric, alternating asymmetric, alternating inphase* and *alternating antiphase*. For the outcome BCC, condition contrasts were: *Constant symmetric, asymmetric* (*with left hand constant*), *asymmetric* (*with right hand constant*), *alternating inphase* and *alternating antiphase*. Beta coefficients, standard errors and t-statistics (with Pearson r as effect size) are reported for planned contrast comparisons.

We have furthermore calculated the intra-subject variability as the standard deviation over all 8 trials. To put the variability between subjects in perspective, we calculated the between subjects standard deviations of the different outcomes and the average of the within subject standard deviations aggregated by group, hand, condition and sex and then calculated a ration score between the two (V_ratio_ = V_between_/V_within_). Scores larger than 1 indicate that between subject variability is greater than within subject variability.

## Results

**FIGURE 4:**
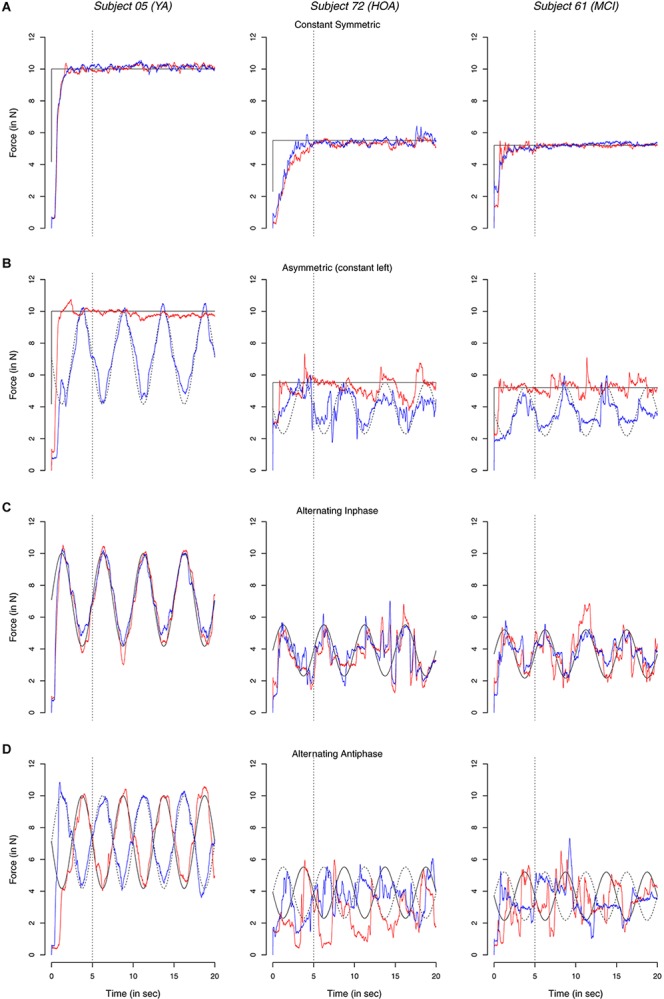
Exemplary signals showing time continuous force dynamics of the non-dominant (red) and dominant hand (blue line) and respective target values (thin dashed and solid lines); dotted vertical lines indicate initial 5 s. Data profiles are shown from three exemplary individuals one from each of the groups, younger adults (YA), healthy older adults (HOA) and older adults with mild cognitive impairments (MCI) in the different conditions.

In Figure 4 exemplary time continuous force profiles in the different conditions are shown for one representative participant in each group (see Supplementary Figure S1 for mean (M) and SD of profiles). Absolute force values of the YA participants were larger than both OA and MCI, resulting from differences in MVC (when comparing groups instead of individuals, however, no significant differences in MVC were found, cf. Table 2). All three individuals display better constant force performance during the *both constant* condition than during the *asymmetric* condition. Overall, this was particularly true in HOA and MCI as compared to YA. Moreover, YA showed better performance during *inphase* and *antiphase* conditions and during symmetric than asymmetric conditions with little deviations to the target line than both HOA and MCI, even more so in the *antiphase* condition.

### Time on Target

**FIGURE 5:**
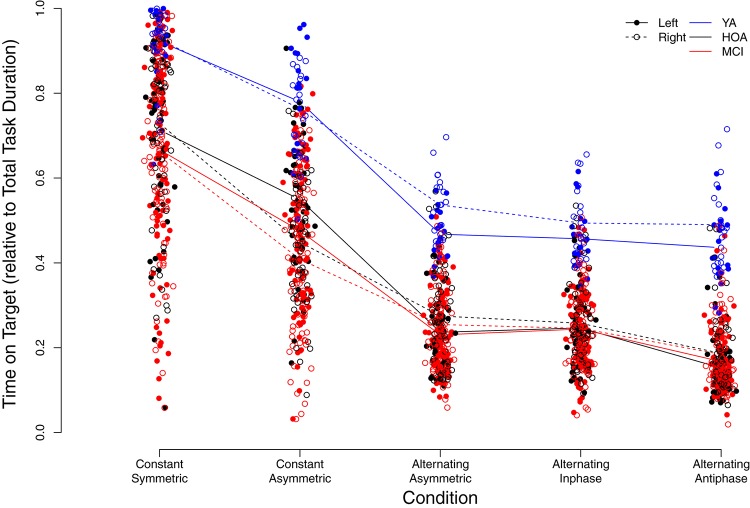
Individual time on target values relative to total task duration (i.e., from 0 = 0% to 1 = 100%) in the different conditions. Trend-lines indicate mean values for the different conditions, groups (YA, younger adults; HOA, Healthy Older Adults; MCI, Older Adults with MCI) as well as left or right hand (see legend for labeling).

Figure 5 displays individual TOT scores for the different conditions with respect to group and hand (see also Supplementary Table S1). Linear mixed model analysis revealed significant main effects for the factors group [*F*(2,158) = 72.32, *p* < 0.001, η_*P*_^2^ = 0.478], with YA showing higher TOT across conditions as opposed to HOA and MCI and HOA as opposed to MCI, condition [*F*(4,636) = 898.98, *p* < 0.001, η_*P*_^2^ = 0.850], with performance deteriorations from constant symmetric to alternating asymmetric, and sex [*F*(1,158) = 25.60, *p* < 0.001, η_*P*_^2^ = 0.139], with lower TOT for females than males, however, not for hand [*F*(1,803) = 0.006, *p* = 0.938, η_*P*_^2^ = 0.000] (cf. Table 3 for planned contrasts).

**TABLE 3:** Parameters of planned contrasts on main and interaction effects for outcome Time on Target (TOT).

		Beta	Std. Error	DF	*t*-Value	*p*-Value	r
**Intercept**		**0.498**	**0.027**	**803**	**18.57**	**<0.001**	**0.55**
***Group***							
**HOA vs. MCI**		**−0.038**	**0.017**	**158**	**−2.30**	**0.023**	**0.18**
**HOA vs. YA**		**0.222**	**0.025**	**158**	**8.82**	**<0.001**	**0.57**
***Condition***							
**Constant Symmetric vs. Constant Asymmetric**		**−0.185**	**0.018**	**636**	**−10.02**	**<0.001**	**0.37**
**Constant Asymmetric vs. Alternating Asymmetric**		**−0.296**	**0.018**	**636**	**−16.10**	**<0.001**	**0.54**
Alternating Asymmetric vs. Alternating Inphase		0.009	0.018	636	0.50	0.620	0.02
**Alternating Inphase vs. Alternating Antiphase**		**−0.092**	**0.018**	**636**	**−4.98**	**<0.001**	**0.19**
***Sex***							
**M vs. F**		**−0.074**	**0.015**	**158**	**−5.06**	**<0.001**	**0.37**
***Group X Condition***							
HOA vs. MCI	Constant Symmetric vs. Constant Asymmetric	0.000	0.022	636	**−**0.02	0.984	0.00
**HOA vs. YA**	**Constant Symmetric vs. Constant Asymmetric**	**0.069**	**0.034**	**636**	**2.05**	**0.041**	**0.08**
**HOA vs. MCI**	**Constant Asymmetric vs. Alternating Asymmetric**	**0.044**	**0.022**	**636**	**2.00**	**0.046**	**0.08**
HOA vs. YA	Constant Asymmetric vs. Alternating Asymmetric	**−**0.026	0.034	636	**−**0.78	0.437	0.03
HOA vs. MCI	Alternating Asymmetric vs. Alternating Inphase	0.006	0.022	636	0.27	0.788	0.01
HOA vs. YA	Alternating Asymmetric vs. Alternating Inphase	**−**0.023	0.034	636	**−**0.69	0.493	0.03
HOA vs. MCI	Alternating Inphase vs. Alternating Antiphase	0.014	0.022	636	0.63	0.530	0.02
**HOA vs. YA**	**Alternating Inphase vs. Alternating Antiphase**	**0.070**	**0.034**	**636**	**2.08**	**0.038**	**0.08**
***Hand X Condition***							
**Left vs. Right**	**Constant Symmetric vs. Constant Asymmetric**	**−0.075**	**0.012**	**803**	**−6.45**	**<0.001**	**0.22**
**Left vs. Right**	**Constant Asymmetric vs. Alternating Asymmetric**	**0.106**	**0.012**	**803**	**9.04**	**<0.001**	**0.30**
**Left vs. Right**	**Alternating Asymmetric vs. Alternating Inphase**	**−0.025**	**0.012**	**803**	**−2.16**	**0.031**	**0.08**
Left vs. Right	Alternating Inphase vs. Alternating Antiphase	0.018	0.012	803	1.55	0.122	0.05
***Hand X Group***							
Left vs. Right	HOA vs. MCI	**−**0.005	0.008	803	**−**0.65	0.518	0.02
**Left vs. Right**	**HOA vs. YA**	**0.030**	**0.013**	**803**	**2.37**	**0.018**	**0.08**

*Beta, linear coefficient; Std. Error, standard error of the beta coefficient; DF, degrees of freedom; r, effect size; HOA, cognitively healthy older adults; MCI, older adults with mild cognitive impairments; YA, younger adults; M, male; F, female. Bold values indicate significant effects at *p* < 0.05.*

Significant interaction effects were found between group and condition [*F*(8,636) = 3.03, *p* = 0.002, η_*P*_^2^ = 0.037], between condition and hand [*F*(4,803) = 25.98, *p* < 0.001, η_*P*_^2^ = 0.115] as well as between group and hand [*F*(2,803) = 4.41, *p* = 0.013, η_*P*_^2^ = 0.011]. Group x condition interaction revealed larger performance deteriorations for HOA as compared to YA when inspecting contrasts between constant symmetric and constant asymmetric as well as between alternating inphase and alternating antiphase. On the other hand, larger performance deteriorations were found for HOA when compared to MCI from *constant asymmetric* to *alternating asymmetric*. For interactions between hand and condition, larger performance deteriorations were found in the right than left hand from *constant symmetric* to *constant asymmetric*. As opposed to that, when comparing *constant asymmetric* to *alternating asymmetric*, improvements in the right hand were found as compared to left. Inspection of the group x hand interaction showed, that YA displayed larger performance differences between hands (with better performance for the right hand) than HOA (cf. Table 3).

### Variability Structure of Force Output

**FIGURE 6:**
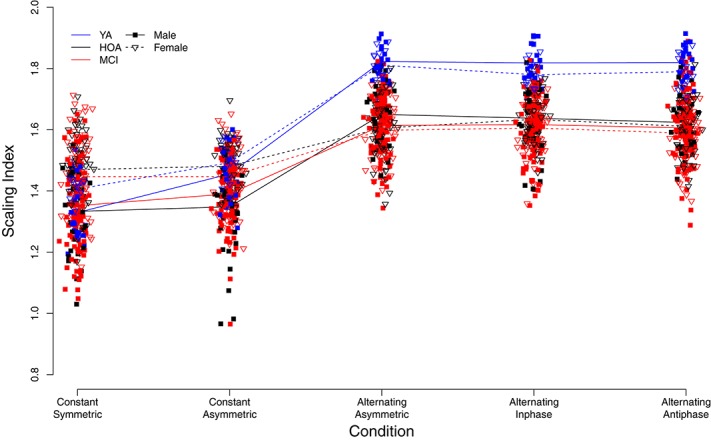
Individual scaling indices derived from detrended fluctuation analysis. Trend lines indicate mean values for each group (YA, younger adults; HOA, Healthy Older Adults; MCI, Older Adults with Mild Cognitive Impairments), sex, and condition (see legend for labeling).

Figure 6 displays individual DFA-α scores for the different conditions, and with respect to group and sex (see also Supplementary Table S2). Similar to TOT, performance variability structure differed across conditions [main effect of condition, *F*(4,632) = 561.32, *p* < 0.001, η_*P*_^2^ = 0.780]. Overall, DFA-α-values for the constant force production task were found to be roughly within the range between pink (α = 1) and Brownian (α = 1.5) noise. Alternating force production tasks yielded an increase in DFA-α toward values of 1.5 and higher, indicating more structured signals with smoother time-series exhibiting slower fluctuations. Furthermore, a group effect was found [*F*(2,158) = 27.83, *p* < 0.001, η_*P*_^2^ = 0.261], with YA showing considerably larger DFA-α during alternating force production as compared to HOA and MCI, and an effect of hand [*F*(1,803) = 8.96, p = 0.003, η_*P*_^2^ = 0.011], with larger DFA-α for the right as compared to the left hand. Furthermore, overall, male participants had smaller exponent values (closer to white noise) than females [main effect of sex, *F*(1,158) = 5.71, *p* = 0.018, η_*P*_^2^ = 0.035].

Sex differences, however, were observed across conditions as indicated by a significant sex by condition interaction [*F*(4,632) = 33.01, *p* < 0.001, η_*P*_^2^ = 0.173]. That is, during constant force production, female participants showed larger exponent values (DFA-α closer to 1.5) than male participants. Male participants, however, displayed larger DFA-α in the alternating force production tasks. The group by condition interaction [*F*(8,632) = 21.65, *p* < 0.001, η_*P*_^2^ = 0.215] showed for YA a lower DFA-α (i.e., closer to 1) in the *constant symmetric* condition and a significantly larger increase of DFA-α (as compared to HOA) in the *constant asymmetric* condition. Group by hand and condition by hand interactions were not significant (cf. Table 4 for planned contrasts).

**TABLE 4:** Parameters of planned contrasts on main and interaction effects for outcome DFA scaling exponent (α).

		Beta	Std. Error	DF	*t*-Value	*p*-Value	r
**Intercept**		**1.525**	**0.012**	**803**	**129.21**	**<0.001**	**0.98**
***Group***							
HOA vs. MCI		**−**0.016	0.012	158	**−**1.33	0.178	0.10
**HOA vs. YA**		**0.111**	**0.019**	**158**	**5.87**	**<0.001**	**0.42**
***Condition***							
Constant Symmetric vs. Constant Asymmetric		0.023	0.017	632	1.39	0.166	0.06
**Constant Asymmetric vs. Alternating Asymmetric**		**0.251**	**0.017**	**632**	**15.16**	**<0.001**	**0.52**
Alternating Asymmetric vs. Alternating Inphase		0.013	0.017	632	0.77	0.442	0.03
Alternating Inphase vs. Alternating Antiphase		**−**0.017	0.017	632	**−**1.03	0.304	0.04
***Hand***							
Left vs. Right		0.005	0.005	803	1.11	0.269	0.04
***Sex***							
**M vs. F**		**0.026**	**0.011**	**158**	**2.39**	**0.018**	**0.19**
***Group X Condition***							
HOA vs. MCI	Constant Symmetric vs. Constant Asymmetric	0.005	0.017	632	0.27	0.785	0.01
**HOA vs. YA**	**Constant Symmetric vs. Constant Asymmetric**	**0.091**	**0.025**	**632**	**3.59**	**<0.001**	**0.14**
HOA vs. MCI	Constant Symmetric vs. Constant Asymmetric	**−**0.020	0.017	632	**−**1.22	0.224	0.05
**HOA vs. YA**	**Constant Asymmetric vs. Alternating Asymmetric**	**0.137**	**0.025**	**632**	**5.42**	**<0.001**	**0.21**
HOA vs. MCI	Alternating Asymmetric vs. Alternating Inphase	**−**0.005	0.017	632	**−**0.32	0.751	0.01
HOA vs. YA	Alternating Asymmetric vs. Alternating Inphase	**−**0.028	0.025	632	**−**1.12	0.263	0.04
HOA vs. MCI	Alternating Inphase vs. Alternating Antiphase	0.005	0.017	632	0.31	0.753	0.01
HOA vs. YA	Alternating Inphase vs. Alternating Antiphase	0.024	0.025	632	0.97	0.335	0.04
***Sex X Condition***							
M vs. F	Constant Symmetric vs. Constant Asymmetric	**−**0.027	0.015	632	**−**1.83	0.068	0.07
**M vs. F**	**Constant Asymmetric vs. Alternating Asymmetric**	**−0.103**	**0.015**	**632**	**−6.82**	**<0.001**	**0.26**
M vs. F	Alternating Asymmetric vs. Alternating Inphase	0.012	0.015	632	0.81	0.416	0.03
M vs. F	Alternating Inphase vs. Alternating Antiphase	**−**0.005	0.015	632	**−**0.35	0.730	0.01

*Beta, linear coefficient; Std. Error, standard error of the beta coefficient; DF, degrees of freedom; r, effect size; HOA, cognitively healthy older adults; MCI, older adults with mild cognitive impairments; YA, younger adults; M, male; F, female. Bold values indicate significant effects at *p* < 0.05.*

Please refer to the Supplementary Material for Figure 2 that displays the log-log plots for average fluctuation functions and the local scaling exponents calculated by use of moving fitting windows to inspect the quality of fitting (c.f., Xia et al., 2013).

### Bimanual Coupling

**FIGURE 7:**
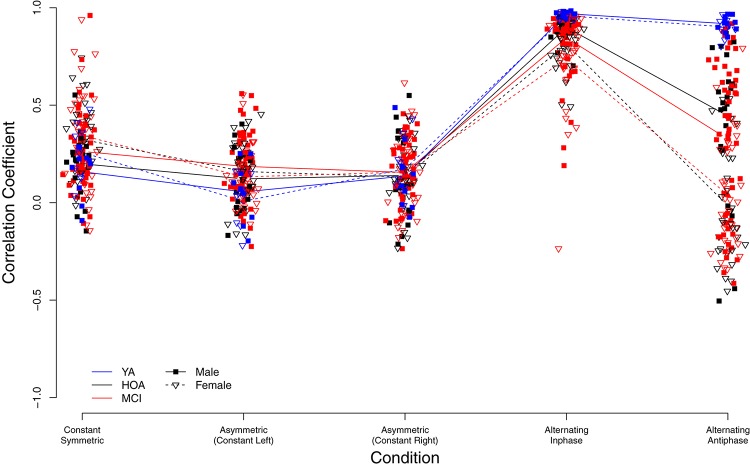
Individual bimanual correlation coefficients (BCC) between left and right hand signals displayed by group (YA, younger adults; OA, Older adults; MCI, Older adults with mild cognitive impairments) and condition.

As shown in Figure 7, higher bimanual coupling scores (BCC) were observed in YA in the two conditions *alternating inphase* and *alternating antiphase* as compared to HOA and MCI. Contrarily, coupling was slightly lower in YA (as compared to both other groups) in the *constant symmetric* and *asymmetric* (*with left hand constant*) conditions, however not in the *asymmetric* (*with right hand constant*) condition. Further, sex differences are obvious and differently pronounced across conditions (cf. Figure 7). Linear mixed effects modeling showed a significant group effect [*F*(2,156) = 14.69, *p* < 0.001, η_*P*_^2^ = 0.159] as well as condition effect [*F*(4,632) = 300.95, *p* < 0.001, η_*P*_^2^ = 0.656]. Furthermore, a main effect for sex was found [*F*(1,156) = 15.78, *p* < 0.001, η_*P*_^2^ = 0.092], differences between male and female participants differed, however, between conditions. A significant interaction effect was found between group and condition [*F*(8,632) = 22.81, *p* < 0.001, η_*P*_^2^ = 0.224] and between sex and condition [*F*(4,632) = 20.45, *p* < 0.001, η_*P*_^2^ = 0.115].

Inspection of planned contrasts (c.f. Table 5) showed lower BCC values in the *constant symmetric* and the two *constant asymmetric* conditions as compared to alternating. Further contrast inspection for the interaction terms showed a significantly larger reduction in BCC for HOA as compared to YA from *alternating inphase* toward *alternating antiphase*. While female participants showed larger BCC values than male in the *constant symmetric* condition, they also showed larger reductions in *the asymmetric* (*constant left*) condition. Furthermore, considerably lower BCC values were shown for female participants in the alternating inphase and alternating antiphase conditions than male participants.

**TABLE 5:** Parameters of planned contrasts on main and interaction effects for outcome bimanual coupling coefficient (BCC).

		Beta	Std. Error	DF	*t*-Value	*p*-Value	r
**Intercept**		**0.439**	**0.049**	**632**	**9.06**	**<0.001**	**0.34**
***Group***							
HOA vs. MCI		**−**0.011	0.058	156	**−**0.20	0.844	0.02
HOA vs. YA		**−**0.004	0.087	156	**−**0.04	0.966	0.00
***Condition***							
Constant Symmetric vs. Asymmetric (constant left)		0.066	0.087	632	0.76	0.448	0.03
Asymmetric (constant left) vs. Asymmetric (constant right)		**−**0.054	0.087	632	**−**0.62	0.535	0.02
**Asymmetric (constant right) vs. Alternating Inphase**		**0.858**	**0.087**	**632**	**9.83**	**<0.001**	**0.36**
**Alternating Inphase vs. Alternating Antiphase**		**−0.329**	**0.087**	**632**	**−3.77**	**<0.001**	**0.15**
Sex							
**M vs. F**		**−0.078**	**0.029**	**156**	**−2.73**	**0.007**	**0.21**
***Group X Condition***							
HOA vs. MCI	Constant Symmetric vs. Asymmetric (constant left)	**−**0.026	0.053	632	**−**0.50	0.617	0.02
HOA vs. YA	Constant Symmetric vs. Asymmetric (constant left)	**−**0.060	0.080	632	**−**0.75	0.456	0.03
HOA vs. MCI	Asymmetric (constant left) vs. Asymmetric (constant right)	0.008	0.053	632	0.15	0.833	0.01
HOA vs. YA	Asymmetric (constant left) vs. Asymmetric (constant right)	0.136	0.080	632	1.70	0.090	0.07
HOA vs. MCI	Asymmetric (constant right) vs. Alternating Inphase	**−**0.084	0.053	632	**−**1.59	0.113	0.06
HOA vs. YA	Asymmetric (constant right) vs. Alternating Inphase	0.086	0.080	632	1.07	0.245	0.04
HOA vs. MCI	Alternating Inphase vs. Alternating Antiphase	0.046	0.053	632	0.87	0.385	0.03
**HOA vs. YA**	**Alternating Inphase vs. Alternating Antiphase**	**0.060**	**0.080**	**632**	**7.50**	**<0.001**	**0.29**
***Sex X Condition***							
**M vs. F**	**Constant Symmetric vs. Asymmetric (constant left)**	**−0.122**	**0.048**	**632**	**−2.57**	**0.011**	**0.10**
M vs. F	Asymmetric (constant left) vs. Asymmetric (constant right)	0.027	0.048	632	0.57	0.572	0.02
M vs. F	Asymmetric (constant right) vs. Alternating Inphase	**−**0.091	0.048	632	**−**1.90	0.057	0.08
**M vs. F**	**Alternating Inphase vs. Alternating Antiphase**	**−0.221**	**0.048**	**632**	**−4.64**	**<0.001**	**0.18**

*Beta, linear coefficient; Std. Error, standard error of the beta coefficient; DF, degrees of freedom; r, effect size; HOA, cognitively healthy older adults; MCI, older adults with mild cognitive impairments; YA, younger adults; M, male; F, female. Bold values indicate significant effects at *p* < 0.05.*

### Intra- vs. Inter-Individual Variability

Comparison of intra- and inter-individual variability shows V_ratio_ scores larger than 1 across outcome measures (TOT: Vratio = 1.51 ± 0.53; DFA-α: Vratio = 1.61 ± 0.45; BCC: Vratio = 1.20 ± 0.48), thus showing larger between than within subject variability.

## Discussion

In this study, we investigated how age, cognitive function and sex affect bimanual force control in tasks that differ with respect to task difficulty (i.e., constant or alternating force production) as well as symmetry. We therefore analyzed different measures reflecting overall performance (i.e., TOT), behavioral complexity as it can be inferred from the time-structure of force control signals (i.e., DFA) and interlimb coupling (i.e., BCC). Due to asymmetries in intra- and interhemispheric information processing, we expected bimanual performance to be differentially affected depending on the executing hand. Furthermore, due to changes in structural integrity, we expected group differences with respect to age, cognitive status and sex.

As expected, we found performance differences between the different conditions that were characterized by differences in difficulty (i.e., constant or alternating) and task symmetry. We also found performance differences between the right and left hand. These differences were, however, task dependent with the left hand showing better performance in the asymmetric task when producing the constant force and the right hand when producing the alternating force, for example. With respect to age, also as expected, we found lower performance (i.e., TOT) in HOA and MCI as compared to YA in all conditions. Furthermore, YA displayed better coupling in *alterating inphase* and *alternating antiphase* likewise suggesting better performance in these conditions. As expected, large differences in variability structure were found between constant and alternating force production tasks with smaller DFA-α exponents (ranging between 1 –pink– and 1.5 –Brownian– noise) in the former task that requires a fixed-point dynamics, than the latter (DFA-α ≥ 1.5) that requires an oscillatory-like dynamics (Knol et al., 2019). Performance differences between HOA and MCI were less clear than expected, however. HOA showed slightly better performance, particularly in the less difficult (constant force production) tasks; difference for coupling and structure of variability could not be shown between HOA and MCI. Unexpectedly, large sex effects were found for the structure of variability and coupling with worse performance of female participants. In the following sections, effects of task symmetry, executing hand, age and cognitive impairment as well as sex are discussed in detail.

### Bimanual Task Symmetry

We investigated bimanual force coordination in symmetric and asymmetric tasks requiring both, constant and alternating force production. Task symmetry was one of the main factors found in this study that affect bimanual performance across the different outcome measures. Performance differences between alternating symmetric and asymmetric (in- and antiphase) tasks have repeatedly been shown in previous studies investigating stability of the relative phase (Haken et al., 1985) with reduced stability in the antiphase condition. Likewise, lower performance has previously been shown during constant force production when the opposite hand simultaneously produced an alternating force (i.e., asymmetric), as compared to a simultaneous constant force production (i.e., symmetric) (Fling and Seidler, 2012b). These performance deteriorations in *asymmetric* and *antiphase* tasks may be caused by interhemispheric cross-talk leading to involuntary activation such as mirror movements (Zielinski et al., 2017). Accordingly, we observed significantly positive coupling between hands for the *asymmetric* task, suggesting that cross-talk is not sufficiently inhibited by most of the participants (from all groups) causing mirror movements even in healthy YA. As opposed to the negative effects of cross-talk, interhemispheric connection also facilitates bimanual coordination since coupling of both hands is often necessary when tight spatiotemporal coordination is required. Essentially, coupling of the two hands reduces the degrees of freedom to be controlled from two (i.e., each hand separately) to one (i.e., both hands in synergy) (Kelso and Schöner, 1988; Latash et al., 2002). A mechanism of this coupling can be a temporal sequencing of muscle commands (Liuzzi et al., 2011). During symmetric bimanual tasks (e.g., *constant symmetric* or *alternating inphase*), homologous muscles in both hands are activated at the same time. During asymmetric coordination patterns (e.g., *alternating antiphase*) coupling of antagonist muscles (e.g., coupling flexor and extensor activity) may also facilitate coordination. In this study, we also used an *asymmetric* task combining a constant and an alternating force production task. Here, decoupling of the hands or active inhibition of cross-talk (Daffertshofer et al., 2005) is important to avoid involuntary activation during constant force production. In this study, *alternating in*- and *antiphase* yielded large coupling values indicating tight temporal coordination. Likewise, the *constant symmetric* condition resulted in BCC larger than 0. This shows, that positive bimanual coupling (i.e., coupling of homologous muscles) may have some strategic importance for this task.

The symmetry of the bimanual task also had an effect on the structure of variability, from which one can infer behavioral complexity, of constant force production in YA. Namely, the fluctuations were less structured and predictable in the *constant symmetric* task (DFA-α closer to 1) than in the *constant asymmetric* task (higher DFA-α). This finding adds to the previously reported results in unimanual constant as compared to alternating force production using the same (Vaillancourt and Newell, 2003) or different metrics (Knol et al., 2019). It suggests that the self-organizing properties of elemental degrees of freedom of the neuromuscular system to adapt to the task take into account the overall constraints imposed on the limbs as a unit. Here, for the first time, we reveal how the expressed behavioral complexity in a bimanual task is affected by the (a)symmetry of the constraints assigned to each limb. The production of a sine-wave force pattern seems to require the formation of lower dimensional synergies of motor units leading to more regular patterns. What can be interpreted as a decreased signal complexity in the constant force production during the asymmetric task may be a result of the cross-talk between hemispheres and matching of the less complex processes of the sine-wave force producing hand (Schloesser et al., 2019). Complexity matching was primarily found in YA, however, not in HOA or MCI (see further discussion on healthy aging cognitive decline).

### Executing Hand

Performance differences between the left and right hand became visible particularly for TOT (Figure 4). While no particular difference between hands were found in the *constant symmetric* condition, an interesting pattern emerged for all groups for the *asymmetric* condition. We observed a better performance for the left over the right hand when performing the *constant* force production task in the asymmetric condition and the reverse when performing the *alternating* task. Lateralization of the hands is assumed to result from asymmetric specialization due to different task demands for the dominant and non-dominant hand (Guiard, 1987; Wang and Sainburg, 2007), which is part of motor development (Rudisch et al., 2018; Scharoun Benson and Bryden, 2019). Typically, during asymmetric tasks, the non-dominant hand preferentially performs the stabilizing part of the movement (as required in our asymmetric task with left hand constant) whereas the dominant hand is used for manipulation (Guiard, 1987) (as required in our asymmetric task with right hand alternating). Hemispheric asymmetries have also been shown in functional magnetic resonance imaging studies, such as an increased activation in the non-dominant hemisphere during complex bimanual movements (Wenderoth et al., 2004; van den Berg et al., 2010), probably as a result (or reason) of this specialization. The non-dominant hemisphere may thus be more specialized than the dominant hemisphere for the purpose of actively inhibiting neural cross talk.

Armatas et al. (1996) have shown that mirror movements are more pronounced when the non-dominant hand is active. Our results are in line with this finding showing performance differences between hands for the *asymmetric* task (i.e., role-differentiated) with better performance when the left hand produced the *constant* and the right hand the *alternating* force. Differences in BCC between *asymmetric* (*constant left*) and *asymmetric* (*constant right*) were also found for YA with BCC close to 0 in *asymmetric* (*constant left*) (Figure 7), indicating decoupling of the hands with no mirror movements. When taking reversed roles, that is when the right hand produces the constant force, an increased BCC suggests coupling of the two hemispheres leading to involuntary simultaneous activation patterns in both hands. The increase in BCC in YA when comparing the two asymmetric tasks (constant left vs. constant right) does provide support for our hypothesis that there is a hemispheric asymmetry in the capability to actively inhibit cross-talk (van den Berg et al., 2010). Our data also show that this asymmetry is reduced with age (cf. below).

In YA, performance differences between the left and right hand (i.e., TOT) were also found between *alternating inphase* and *alternating antiphase* condition with better performance in the right hand. Interestingly, this was despite the high amount of coupling between hands for YA in the two mentioned conditions. Inspection of individual data points (open blue circles in Figure 4) shows, however, that these differences may be caused by a number of individuals that display particularly good performance with their right hand in the YA group. One possible explanation for this result may therefore be that these individuals follow a strategy of focusing on the force production of the right hand (i.e., coupling with the target) and disregard the opposing hand to enhance the preferred limb performance.

### Healthy Aging and Cognitive Decline

Across conditions, significantly lower TOT was found for HOA and MCI when compared to YA. Furthermore, poorer performance was found for MCI compared to HOA, particularly in tasks requiring constant force production. These results are in line with previous studies on unimanual tasks showing age differences between younger and older adults while performing constant (Galganski et al., 1993) and alternating (Vieluf et al., 2013) force production tasks. Possible mechanisms mediating these effects could be age-related differences in tactile perception (Reuter et al., 2012) and neuromotor control as expressed by motor unit discharge patterns (Vaillancourt et al., 2003). Inspection of interaction effects for TOT showed that performance differences between groups were larger in some conditions than others. HOA and MCI showed larger performance deteriorations compared to YA from *constant symmetric* to *constant asymmetric*. Performance differences between *constant asymmetric* and *alternating asymmetric* were smaller, however, in MCI as compared to HOA. Differences between YA and the two older groups were also found for bimanual coupling with both groups of OA showing high coupling in the *alternating inphase* condition, and HOA as well as MCI (but not YA) showing poorer coupling in *alternating antiphase*. Loss of stability (measured by the relative phase) in antiphase coordination patterns (followed by sudden changes toward inphase) has previously been shown at a critical frequency (Haken et al., 1985) that was found to decrease with aging (Temprado et al., 2010), or when adding secondary cognitive task demands (Temprado et al., 2001). In our study, older adults likewise showed poorer performance in antiphase coordination. Frequency of our sine-wave task was 0.2 Hz and thus, compared to other studies, rather low. Hence, *alternating antiphase* performance was probably as easy to maintain for YA than *alternating inphase.* Contrary, both, HOA and MCI showed large performance reductions in *alternating antiphase* when compared to *alternating inphase*, however, emphasizing the difficulties of older adults to maintain antiphase coordination patterns.

As pointed out above, previous studies have already shown that asymmetric (Fling and Seidler, 2012a) and antiphase (Temprado et al., 2010) bimanual coordination is impaired in older adults, without, however, investigating the impact of cognitive status. Possible mechanisms for the deterioration of bimanual coordination are: (i) a lack of coupling as a result of reduced connectivity between hemispheres (O’Sullivan et al., 2001); or (ii) an increase in interhemispheric interference following a loss in the ability to actively inhibit cross-talk between hemispheres (Daffertshofer et al., 2005). Typically, cross-talk is actively inhibited by inter- and intracortical networks including M1, PMC, and SMA to reduce or eliminate involuntary activation causing mirror movements (Daffertshofer et al., 2005; Grefkes et al., 2008). With aging, on the one hand, the inhibition of cross-talk might be impaired, but on the other hand cross-task might be generally weaker. Different studies have reported on the effect of aging and age-related cognitive impairment on interhemispheric connectivity (O’Sullivan et al., 2001; Frederiksen, 2013; Stricker et al., 2016) and inhibition of cross-talk. For example, Fling and Seidler (2012a) found that YA showed higher integrity of interhemispheric connections as compared to HOA from analysis of diffusion tensor imaging. Results derived from TMS in the same study were less clear, only suggesting a trend relationship between age and interhemispheric connectivity. Tsutsumi et al. (2012) on the other hand found a significant reduction of interhemispheric inhibition in MCI as compared to HOA when using TMS.

Due to the neurophysiological results by Tsutsumi et al. (2012), we expected to find bimanual force control to be affected by cognitive status of older adults, with reduced interhemispheric connectivity leading to reduced bimanual coupling in MCI. As compared to HOA, MCI, however, only showed a trend toward reduced coupling *in alternating inphase*, which may be an indicator of impaired interhemispheric information processing of MCI participants. On the other hand, both HOA and MCI were strongly affected by the *alternating antiphase* condition with significantly lower antiphase coupling than YA, suggesting that YA can easily stabilize the antiphase oscillatory bimanual coordination pattern at the low frequency as provided in this study. Possibly due to impaired intra- and interhemispheric information processing HOA and MCI on the other hand have substantial problems in maintaining antiphase coordination patterns. All groups displayed similar BCC values larger than 0 (see Figure 6) in the *asymmetric* (*constant right*) condition, suggesting insufficient inhibition of cross-talk (Daffertshofer et al., 2005). However, YA showed better coupling (i.e., BCC values closer to 0) in the *asymmetric* (*constant left*) condition than HOA and MCI. Performance differences between conditions are discussed above with respect to lateralization and the division of labor in asymmetric bimanual tasks. For both, HOA and MCI, differences between the two asymmetric conditions disappeared, however, which may be due to a reduction of hemispheric asymmetries in specialized bimanual coordination networks. Hemispheric asymmetries for specialized neural networks that serve other neurocognitive functions, such as memory, have also been shown to disappear with age (Cabeza, 2002). This hemispheric asymmetry reduction in older adults may also be reflected in networks that actively inhibit motor cross-talk between hemispheres.

In addition to TOT and BCC, we also investigated the DFA-α as a proxy of behavioral complexity of force production in the different tasks. Previous studies have shown that expressed complexity of human physiological systems such as the motor (Vaillancourt and Newell, 2002; Hausdorff, 2007; Sleimen-Malkoun et al., 2014) or cardiovascular system (Lipsitz and Goldberger, 1992; Ivanov et al., 1999; Ivanov et al., 2001) can deteriorate with age and disease. Changes of complexity can occur on the level of the number of functional units, such as neurons that make up neural networks, or the number of connections between them. Despite the apparent link to outcome variability, complexity in human physiology is also extremely important for the system to maintain stability when confronted with variable environmental or task-related situations, noise or physiological stress (Lipsitz and Goldberger, 1992). It is thus assumed that following a loss of complexity (Lipsitz and Goldberger, 1992), the human sensorimotor system loses its ability to flexibly adjust to noise and varying task demands, resulting in a reduction of performance stability. Sources of noise in human sensorimotor control can be inherent on the different levels of information processing (Faisal et al., 2008) or due to external perturbations, changing environment or task demands. Stochastic fluctuations observed in different behavioral and physiological processes are a marker of systems complexity and have been shown to change with age (Goldberger et al., 2002b; Vaillancourt and Newell, 2003). For unimanual force production, Vaillancourt and Newell (2003) showed lower DFA-α in constant and larger DFA-α in unimanual sine-wave force production. Furthermore, in their study YA displayed lower DFA-α than HOA for the constant force production and higher DFA-α in the sine-wave task (Vaillancourt and Newell, 2003). This is in line with our results in the alternating force conditions where YA showed significantly larger DFA-α values than HOA and MCI. Contrary however, for the *constant symmetric* task, we found no statistically significant differences between groups. Apart from the bimanual nature, our task required pinch grip force production with the thumb and index finger, while that of Vaillancourt and Newell (2003) required participants to produce a force by pressing the lateral side of their index finger against a force transducer. Their task allowed less precise control as less motor units were involved. Hence, neuromuscular structural deteriorations that occur with age may have a larger effect on the complexity of force production.

Strikingly, when performing the constant task in the asymmetric condition, we found a significant DFA-α increase in YA, but not in HOA or MCI. We hypothesize, that the hand performing the alternating regular pattern entrained the one performing the constant task (via bimanual coupling) and thus restricted its degrees of freedom. We interpret this result as further evidence for a reduced interhemispheric connectivity caused by structural deteriorations of the interhemispheric connections (i.e., CC) which is present in OA and more pronounced in subjects with MCI (Frederiksen, 2013).

### Sex Differences

Unexpectedly, we observed strong interaction effects of task condition and sex on DFA-α and bimanual coupling. Although it was anticipated that sex had an impact on performance, effects were larger than expected.

Previous studies, focusing on unimanual tasks, have reported higher dexterity in older females as compared to males (Ranganathan et al., 2001; Vasylenko et al., 2018). These differences have mostly been attributed to different lifestyles with females more often engaging in work that practices fine motor control (e.g., needlework). Ranganathan et al. (2001) have shown, however, that older men produced considerably higher pinch grip forces than older women. At the same time, older women displayed larger variability in a constant force production task. As measure of variability (or rather of steadiness), Ranganathan et al. (2001) calculated the standard deviation of the signal which is rather related to our measure of task performance (TOT) as it expresses the deviation from a fixed value (e.g., the target value). In that, we likewise found poorer performance in female as compared to male participants (i.e., lower TOT, see Table 3). In our study, we additionally investigated the variability structure of force production, with female participants displaying more predictable, thus presumably, less complex constant force production. By combining the two means of analysis, it becomes apparent that, along with their reduced accuracy, female participants showed a less complex (more regular) force output in constant force production tasks.

Studies comparing bimanual performance between older male and female participants are sparse, yet, in contrast to unimanual dexterity, those that were identified have reported a male advantage (Shetty et al., 2014). Investigation of differences in interhemispheric connectivity in adults between 20 and 80 years of age has shown differential effects of age and sex on microstructural integrity, depending on the observed region (Sullivan et al., 2010). For the genu, the premotor area and the splenium of the CC, significant age-related reductions have been shown for women, however not for men (Sullivan et al., 2010). The different regions play different roles in interhemispheric information processing with respect to bimanual coordination. In our study, older female participants showed particularly poor bimanual coupling in *alternating inphase* and *alternating antiphase* condition. On the other hand, female participants showed higher coupling in the *constant symmetric* condition and a significant decrease in coupling in the *asymmetric* (*constant left*) condition when compared to male participants. These results indicate that older females have less interhemispheric coupling due to the reduced connectivity which, in turn, also results in a reduction of interhemispheric interference in asymmetric tasks.

### Limitations

One possible limitation of this study may be the screening of MCI (Nasreddine et al., 2005). While the MoCA has been reported to have reasonable sensitivity and specificity for the detection of MCI (ca. 90%), it is not as precise as a genuine clinical assessment. More than 50% of the participants scored between 24 and 28 on the MoCA (see Figure 1). Using a cut-off score of <27, this range may be particularly prone to identify false positives or negatives. Further, some of the effects between conditions found in this study may be attributed to fatigue or practice. Counter-balanced designs should be used in future studies that investigate these effects in more detail. Finally, in this study, we have tested the effects of age and cognitive impairment on bimanual control and have discussed these in light of potential structural and functional changes in the brain which we have not directly tested. Further inclusion of neurophysiological measures, e.g., to relate behavioral outcomes to structural integrity or functional connectivity, may help explaining a larger proportion of the variance of our results.

## Conclusion

In sum, we could show that bimanual force control shows differential relations to age, cognitive status and sex when investigating different outcome measures. Differences in intra- and interhemispheric information processing due to healthy aging, cognitive decline, as well as sex have differential effects that depend on task symmetry, but also the specific measures (and processes) under investigation. Overall, we found strong effects of age, executing hand, task symmetry and sex as well as interactions between these factors. That is HOA and MCI generally showed reduced performance across tasks when compared to YA. Furthermore, more complex tasks requiring active interhemispheric inhibition (e.g., such as antiphase coordination patterns or asymmetric tasks) are more affected by age. Unexpectedly, however, large sex effects were found that indicate differences in the coupling between hemispheres between men and women. Contrary to our expectations, the variety of measures only showed small or no differences between HOA and MCI. Therefore, behavioral differences are difficult to interpret with respect to our initial aim to use markers of bimanual force control as prodromal markers of age-related cognitive decline. Deteriorated interhemispheric connectivity that has been shown with MCI was expected to reduce the amount of interhemispheric interference and therefore possibly reduce mirror movements in asymmetric tasks. However, additional changes in information processing (e.g., reduced capacity of active inhibition) may be associated with MCI and therefore diminish this effect. Future studies are therefore needed to investigated neural correlates and decompose intra- and interhemispheric information processing in these different tasks and with respect to populations with structural impairment.

## Data Availability Statement

The datasets generated for this study are available on request to the corresponding author.

## Ethics Statement

The studies involving human participants were reviewed and approved by the Faculty of Behavioural and Social Sciences Research Ethics Committee, Chemnitz University of Technology (V-232-17-KM-SENDA-07112017). The participants provided their written informed consent to participate in this study.

## Author Contributions

JR, KM, LB, DK, and CV-R contributed conception and design of the study. LB and KM collected the data. RS-M contributed analysis tools. JR performed the analysis and wrote the first version of the manuscript. All authors contributed to manuscript revision, read, and approved the submitted version.

## Conflict of Interest

The authors declare that the research was conducted in the absence of any commercial or financial relationships that could be construed as a potential conflict of interest.

## Supplementary Material

FIGURE S1 Average force values (solid lines) and standard deviations (shaded areas) for all participants of YA (blue), HOA (black) and MCI (red) respective for the different conditions as well as the left and right hand.

FIGURE S2 Plots of average detrended fluctuation functions [F(s)] for different conditions, hands and groups (YA, blue; HOA, black; MCI, red) with respect to different window sizes (s). Both, fluctuation functions and window sizes are plotted on logarithmic scales (base 10).

FIGURE S3 Spectrum of local scaling exponents [α(s)] with respect to window sizes. Local scaling exponents were calculated over moving fitting windows of 5 consecutive data points (c.f., Xia et al., 2013). Window sizes (s) are plotted on a logarithmic scale (base 10).

TABLE S1 Mean (SD) Time on Target (relative to total task duration with 0 = 0 % and 1 = 100 %) by condition, hand and group.

TABLE S2 Mean (SD) DFA scaling indices by condition, sex, and group.

TABLE S3 Mean (SE) BCC by condition, sex, and group.
