# Supplementary material for: How Age, Cognitive Function and Gender Affect Bimanual Force Control
Source: Front Physiol. 2020 Mar 27;11:245. doi: 10.3389/fphys.2020.00245 (PMC7121519; doi:10.3389/fphys.2020.00245)
Supplement: TABLE S1 — Mean (SD) Time on Target (relative to total task duration with 0 = 0 % and 1 = 100 %) by condition, hand and group. [file Table_1.DOCX]

| *Supplementary Table 1: Mean (SD) Time on Target (relative to total task duration with 0=0% and 1=100%) by condition, hand and group* | | | | |
| --- | --- | --- | --- | --- |
| **Condition** | **Hand** | **YA** | **HOA** | **MCI** |
| Constant Symmetric | Left | 0.922 (0.092) | 0.713 (0.205) | 0.667 (0.237) |
| Constant Symmetric | Right | 0.927 (0.078) | 0.729 (0.2) | 0.663 (0.215) |
| Constant Asymmetric | Left | 0.78 (0.135) | 0.549 (0.152) | 0.476 (0.177) |
| Constant Asymmetric | Right | 0.764 (0.089) | 0.449 (0.147) | 0.409 (0.172) |
| Alternating Asymmetric | Left | 0.468 (0.058) | 0.236 (0.088) | 0.23 (0.092) |
| Alternating Asymmetric | Right | 0.536 (0.076) | 0.275 (0.114) | 0.255 (0.098) |
| Alternating Inphase | Left | 0.457 (0.074) | 0.247 (0.093) | 0.244 (0.092) |
| Alternating Inphase | Right | 0.494 (0.086) | 0.258 (0.101) | 0.246 (0.097) |
| Alternating Antiphase | Left | 0.436 (0.088) | 0.152 (0.066) | 0.167 (0.078) |
| Alternating Antiphase | Right | 0.49 (0.102) | 0.186 (0.091) | 0.184 (0.088) |
| YA = Young Adults; HOA = Healthy Older Adults; MCI = Older Adults with Mild Cognitive Impairments | | | | |

| *Supplementary Table 2: Mean (SD) DFA scaling indices by condition, sex and group* | | | | |
| --- | --- | --- | --- | --- |
| **Condition** | **Sex** | **YA** | **HOA** | **MCI** |
| Constant Symmetric | Male | 1.329 (0.093) | 1.333 (0.119) | 1.352 (0.134) |
| Constant Symmetric | Female | 1.406 (0.067) | 1.469 (0.11) | 1.447 (0.131) |
| Constant Asymmetric | Male | 1.456 (0.093) | 1.348 (0.132) | 1.391 (0.102) |
| Constant Asymmetric | Female | 1.493 (0.06) | 1.481 (0.077) | 1.447 (0.1) |
| Alternating Asymmetric | Male | 1.823 (0.048) | 1.65 (0.096) | 1.614 (0.097) |
| Alternating Asymmetric | Female | 1.811 (0.046) | 1.605 (0.097) | 1.598 (0.085) |
| Alternating Inphase | Male | 1.818 (0.058) | 1.637 (0.099) | 1.617 (0.1) |
| Alternating Inphase | Female | 1.78 (0.044) | 1.634 (0.077) | 1.606 (0.076) |
| Alternating Antiphase | Male | 1.82 (0.055) | 1.624 (0.088) | 1.606 (0.105) |
| Alternating Antiphase | Female | 1.79 (0.055) | 1.611 (0.082) | 1.589 (0.084) |
| YA = Young Adults; HOA = Healthy Older Adults; MCI = Older Adults with Mild Cognitive Impairments | | | | |

| *Supplementary Table 3: Mean (SD) BCC by condition, sex and group* | | | | |
| --- | --- | --- | --- | --- |
| **Condition** | **Sex** | **YA** | **HOA** | **MCI** |
| Constant Symmetric | Male | 0.159 (0.128) | 0.199 (0.158) | 0.263 (0.189) |
| Constant Symmetric | Female | 0.258 (0.15) | 0.325 (0.189) | 0.345 (0.249) |
| Asymmetric (Constant Non-Dom) | Male | 0.055 (0.149) | 0.122 (0.155) | 0.186 (0.171) |
| Asymmetric (Constant Non-Dom) | Female | 0.007 (0.128) | 0.16 (0.159) | 0.132 (0.144) |
| Asymmetric (Constant Dom) | Male | 0.136 (0.171) | 0.14 (0.213) | 0.155 (0.159) |
| Asymmetric (Constant Dom) | Female | 0.173 (0.145) | 0.131 (0.174) | 0.157 (0.188) |
| Alternating Inphase | Male | 0.97 (0.013) | 0.899 (0.065) | 0.846 (0.165) |
| Alternating Inphase | Female | 0.958 (0.017) | 0.813 (0.114) | 0.74 (0.227) |
| Alternating Antiphase | Male | 0.918 (0.04) | 0.444 (0.383) | 0.326 (0.401) |
| Alternating Antiphase | Female | 0.901 (0.054) | -0.017 (0.287) | 0.039 (0.28) |
| YA = Young Adults; HOA = Healthy Older Adults; MCI = Older Adults with Mild Cognitive Impairments | | | | |
